# Integrative Analysis Coupled with In Vitro Validation Reveals *KAZN* and *SUPT3H* as Shared Negative Regulators in Osteosarcopenia

**DOI:** 10.3390/ijms27146340

**Published:** 2026-07-16

**Authors:** Jiacong Hong, Xiaoyan Zhang, Ganggang Kong, Bailing Chen, Shengli Zhao

**Affiliations:** 1 School of Basic Medical Sciences, Henan Medical University, Xinxiang 453003, China; hongjc@mail3.sysu.edu.cn; 2 Department of Spine Surgery, The First Affiliated Hospital, Sun Yat-sen University, Guangzhou 510080, China; zhangxy359@mail.sysu.edu.cn (X.Z.); konggg@mail.sysu.edu.cn (G.K.)

**Keywords:** osteosarcopenia, sarcopenia, osteoporosis, *KAZN*, *SUPT3H*

## Abstract

Osteosarcopenia, which is the coexistence of sarcopenia and osteoporosis, is being increasingly recognized as a systemic musculoskeletal aging syndrome. However, shared molecular regulators of bone–muscle deterioration remain unclear. In this study, we integrated bulk transcriptomic datasets from sarcopenic skeletal muscle and osteoporosis peripheral blood mononuclear cells to identify shared differentially expressed genes, followed by two-sample Mendelian randomization using osteoporosis genome-wide association study summary statistics to prioritize genes with potential causal relevance. Furthermore, diagnostic performance, functional enrichment, immune infiltration, single-cell RNA sequencing, regulatory network reconstruction, compound prediction, and siRNA-mediated validation were conducted in C2C12 and MC3T3-E1 cells. Overall, 122 shared differentially expressed genes were preliminarily screened, and *KAZN* and *SUPT3H* were tentatively proposed as candidate genes genetically associated with osteoporosis risk. Both genes were upregulated in the disease groups and exhibited weak to modest diagnostic performance. Furthermore, *Kazn* or *Supt3h* knockdown promoted myogenic differentiation in C2C12 cells as well as osteogenic differentiation and mineralization in MC3T3-E1 cells, supporting their roles as negative regulators of lineage differentiation. Moreover, functional analyses linked *KAZN* mainly to mitochondrial-related programs and *SUPT3H* to immune signaling, whereas single-cell analyses localized these genes to stromal, progenitor, and immune-related compartments. These hypothesis-generating findings suggest that *KAZN* and *SUPT3H* participate in shared bone–muscle dysfunction and generate candidate genes and mechanistic hypotheses for subsequent functional validation and translational research.

## 1. Introduction

Sarcopenia and osteoporosis are serious age-related musculoskeletal disorders as well as major contributors to disability, fractures, loss of independence, and reduced quality of life in older adults [1,2]. These two conditions often coexist, a clinical phenotype increasingly known as osteosarcopenia [3,4]. The coexistence of sarcopenia and osteoporosis is clinically relevant, because patients with osteosarcopenia often exhibit a higher risk of falls, fractures, functional impairment, and mortality than those with either condition alone [5]. Epidemiological observations further suggest bidirectional links between muscle and bone loss, thus supporting the concept that osteosarcopenia reflects shared biological mechanisms rather than simply the aggregation of two common geriatric diseases [6].

Interestingly, the muscle–bone axis provides a conceptual framework for understanding this comorbidity. Beyond mechanical loading, skeletal muscles and bones communicate via endocrine, paracrine, metabolic, and inflammatory mediators that affect tissue remodeling and repair [7,8]. From a geroscience perspective, osteosarcopenia may represent a system-level manifestation of musculoskeletal aging in which chronic low-grade inflammation, immune remodeling, mitochondrial dysfunction, and impaired regenerative capacity act collectively to undermine muscle and bone homeostasis [9]. Mitochondrial dysfunction is especially relevant as it contributes to age-related declines in muscle performance and possibly impairs osteogenic capacity and skeletal remodeling [9,10,11]. Thus, identifying shared molecular determinants that connect immune dysregulation with mitochondrial vulnerability could elucidate the biological basis of bone–muscle crosstalk and support the development of better biomarkers and therapeutic hypotheses.

Transcriptome-wide studies have given valuable insights into sarcopenia and osteoporosis by identifying differentially expressed genes and enriched pathways within a single tissue or disease context [12,13]. However, such approaches have limitations when applied to osteosarcopenia. First, single-disease analyses do not definitively prioritize convergent signals shared across muscle and bone-related compartments. Second, association-based transcriptomic signatures cannot reliably distinguish causal drivers from the downstream consequences of disease, medication use, comorbidities, or lifestyle factors. Therefore, establishing causal relevance is crucial if transcriptomic findings are to be translated into robust biomarkers and therapeutic targets, especially for complex age-related syndromes in which confounding and reverse causation are common.

Mendelian randomization (MR) can strengthen causal inference by leveraging genetic variants as instrumental variables, thus reducing confounding and limiting reverse causation in exposure–outcome relationships [14]. When combined with transcriptomic signals, MR provides a principled approach to prioritizing genes with genetic evidence consistent with a causal contribution to disease risk. Regardless, causal prioritization alone is insufficient for defining biological function in a multicellular system. Moreover, cellular localization and differentiation contexts are often required to connect candidate genes to the relevant compartments involved in aging, regeneration, and tissue remodeling. Single-cell RNA sequencing (scRNA-seq) can provide this resolution by mapping prioritized genes to defined cell populations and cell states, including bone marrow mesenchymal stromal cells, satellite cells, fibroadipogenic progenitors, and immune-related compartments [15].

In this study, we integrated cross-tissue transcriptomic profiles from sarcopenic skeletal muscle and osteoporosis peripheral blood mononuclear cells (PBMCs) to identify shared molecular signals. Subsequently, two-sample MR was implemented to prioritize genes with genetic evidence for an association with osteoporosis risk. Candidate genes were subsequently evaluated for expression patterns, diagnostic performance, and biological function. Importantly, we introduced in vitro siRNA-mediated validation to determine if the prioritized genes regulate myogenic and osteogenic differentiation. Subsequent enrichment, immune infiltration, single-cell, regulatory network, and compound prediction analyses were used to categorize the validated genes into broader mechanistic contexts. Collectively, this analytical strategy aimed to preliminarily screen potential shared molecular regulators and generate testable mechanistic hypotheses for osteosarcopenia.

## 2. Results

### 2.1. Cross-Tissue Differential Gene Expression Analysis and MR Prioritize KAZN and SUPT3H

**Figure 1:**
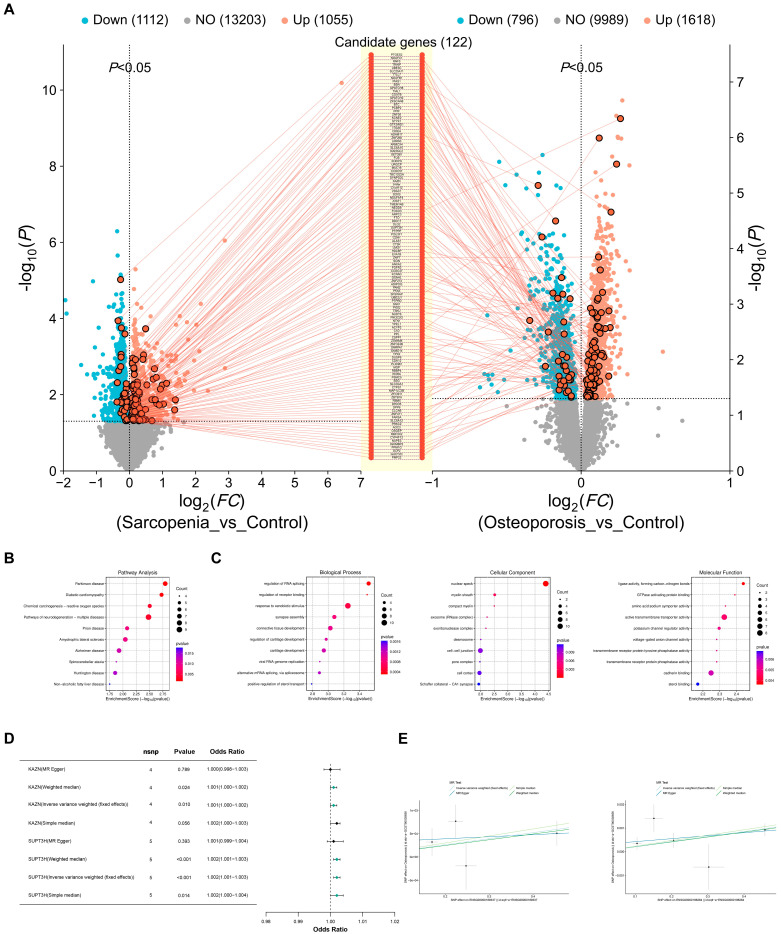
Cross-tissue DEG identification and MR-based prioritization of key genes in sarcopenia and osteoporosis. (**A**) Integrated volcano plot summarizing differential expression results and highlighting the 122 shared DEGs between sarcopenia skeletal muscle samples from GSE111016 and osteoporosis PBMC samples from GSE56815. (**B**) KEGG enrichment bubble plot exhibiting the top enriched pathways for shared DEGs. (**C**) GO enrichment bubble plots showing enriched terms across biological processes, cellular components, and molecular function categories. (**D**) Forest plot of two-sample MR estimates for associations between candidate genes and osteoporosis risk. (**E**) Scatter plots depicting SNP-specific MR estimates for *KAZN* (left) and *SUPT3H* (right). DEG, differentially expressed gene; MR, Mendelian randomization; KEGG, Kyoto Encyclopedia of Genes and Genomes; GO, gene ontology; SNP, single-nucleotide polymorphism.

Differential expression analysis identified 2167 differentially expressed genes (DEGs) in the sarcopenia dataset GSE111016, including 1055 upregulated and 1112 downregulated genes. In the osteoporosis dataset GSE56815, 2414 DEGs were identified, including 1618 upregulated and 796 downregulated genes. Integration of the two disease datasets identified 122 shared DEGs, which were defined as the initial candidate genes for investigating the molecular mechanisms potentially shared between sarcopenia and osteoporosis (Figure 1A).

Functional enrichment analysis was performed to characterize these shared DEGs. Kyoto Encyclopedia of Genes and Genomes (KEGG) enrichment suggested the involvement of neurodegeneration-related pathways, including Parkinson’s disease and amyotrophic lateral sclerosis (Figure 1B). Furthermore, gene ontology (GO) enrichment indicated that the top enriched terms included biological processes such as regulation of RNA splicing, response to xenobiotic stimulus, and connective tissue development; cellular components such as nuclear speck, myelin sheath, and cell–cell junction; and molecular functions such as active transmembrane transporter activity and cadherin binding (Figure 1C). These analyses provided an initial overview of the functional landscape of the shared transcriptomic signature.

To evaluate whether candidate genes showed genetic evidence for an association with osteoporosis risk, we performed two-sample MR. Cis-expression quantitative trait locus (eQTL) instruments were available for 89 of the 122 shared DEGs from OpenGWAS, whereas 33 genes were excluded because eligible instruments were unavailable. Univariable MR screening tentatively proposed *KAZN* and *SUPT3H* as genes whose genetically proxied expression may show a positive correlative signal with osteoporosis risk, and these genes were therefore defined as key candidate genes for downstream analyses (Figure 1D).

Scatter plots showed positive slopes for *KAZN* and *SUPT3H*, consistent with increased osteoporosis risk in association with genetically proxied gene expression (Figure 1E). Heterogeneity tests using the inverse variance weighted (IVW) method showed *p* > 0.05, indicating no significant heterogeneity. Horizontal pleiotropy tests were also nonsignificant for *KAZN* and *SUPT3H* (Table S1). Owing to the limited number of instrumental single-nucleotide polymorphisms (SNPs, four for *KAZN* and five for *SUPT3H*), the MR-Egger sensitivity analysis possessed low statistical power and could not be regarded as a robust test for horizontal pleiotropy. Steiger directionality tests supported the direction from genetically proxied gene expression to osteoporosis risk (Table S2). Collectively, these results prioritized *KAZN* and *SUPT3H* as shared candidate genes with MR support in the osteoporosis context.

### 2.2. Expression Patterns and Diagnostic Performance of KAZN and SUPT3H Across Sarcopenia and Osteoporosis

**Figure 2:**
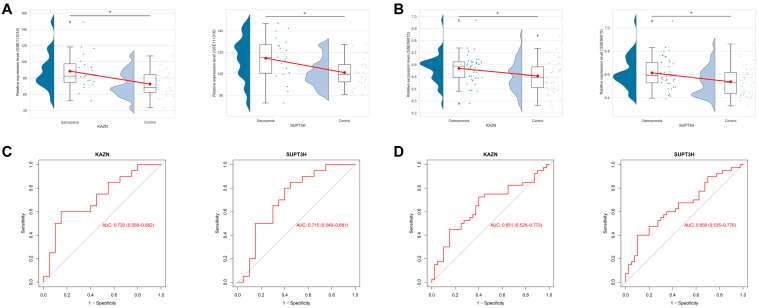
Clinical relevance and diagnostic potential of *KAZN* and *SUPT3H* in sarcopenia and osteoporosis. (**A**) Raincloud plots depicting the expression of *KAZN* and *SUPT3H* in sarcopenia versus control samples in GSE111016. * *p* < 0.05. (**B**) Raincloud plots showing the expression of *KAZN* and *SUPT3H* in low-BMD versus high-BMD samples in GSE56815. * *p* < 0.05. (**C**) In-sample ROC curves evaluating the diagnostic performance of *KAZN* and *SUPT3H* for sarcopenia in GSE111016. (**D**) In-sample ROC curves evaluating the diagnostic performance of *KAZN* and *SUPT3H* for osteoporosis status in GSE56815. BMD, bone mineral density; ROC, receiver operating characteristic.

*KAZN* and *SUPT3H* expression patterns were then evaluated in the original disease datasets. Raincloud plots showed that *KAZN* and *SUPT3H* were significantly upregulated in sarcopenia samples compared with controls in GSE111016 (Figure 2A). Similarly, both genes were upregulated in the osteoporosis dataset, represented by the low bone mineral density (BMD) group compared with the high-BMD group in GSE56815 (Figure 2B).

In-sample receiver operating characteristic (ROC) analysis was performed to assess the diagnostic performance of the two genes. In the sarcopenia cohort, *KAZN* and *SUPT3H* demonstrated modest discriminatory performance, with area under the curve (AUC) values exceeding 0.7 (Figure 2C). In the osteoporosis cohort, both genes showed only a weak trend of between-group differentiation in the internal training dataset, with AUC values exceeding 0.6 (Figure 2D).

To rule out dataset-specific noise and validate the reproducibility of the gene upregulation signals, we validated *KAZN* and *SUPT3H* expression patterns in the independent sarcopenia skeletal muscle dataset GSE167186 and the osteoporosis bone marrow-derived mesenchymal stem cells (BMSCs) dataset GSE249471, respectively.

In the sarcopenia validation cohort, the *KAZN* and *SUPT3H* expression levels in sarcopenia patients followed the same upward trend as in the original discovery set. The expression difference for *KAZN* reached statistical significance, while *SUPT3H* showed a nonsignificant upward trend (Figure S1A). ROC analysis in this cohort yielded AUC values of 0.701 for *KAZN* and 0.582 for *SUPT3H* (Figure S1B).

In the osteoporosis BMSC validation cohort, both genes showed a consistent upward trend in the osteoporosis group compared with controls, but neither difference reached statistical significance due to the limited sample size (Figure S1C). ROC analysis yielded AUC values of 0.889 for *KAZN* and 0.778 for *SUPT3H* (Figure S1D); however, these estimates should be interpreted cautiously, given the small cohort size.

The positive replication of *KAZN* in the tissue-matched sarcopenia cohort indicates that high *KAZN* expression is a reproducible transcriptomic feature in sarcopenic muscle rather than a random fluctuation specific to the GSE111016 dataset. For *SUPT3H* and the osteoporosis signature, partial replication may indicate that these signals are more susceptible to population heterogeneity, tissue context, and sample size limitations, which further underscores the exploratory and hypothesis-generating nature of the current findings.

### 2.3. Experimental Validation Supports Negative Regulatory Roles of Kazn and Supt3h in Myogenic and Osteogenic Differentiation

**Figure 3:**
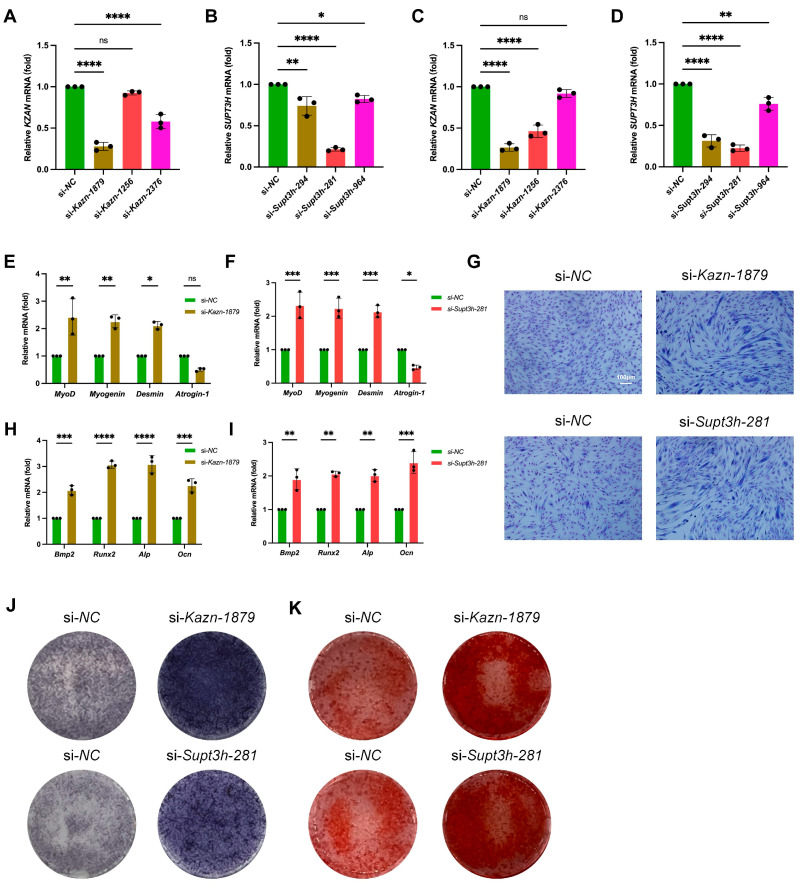
Experimental validation of *Kazn* and *Supt3h* in myogenic and osteogenic differentiation. (**A**–**D**) Knockdown efficiency of *Kazn* and *Supt3h* following siRNA transfection in C2C12 and MC3T3-E1 cells, as assessed by qPCR. (**E**,**F**) qPCR analysis of myogenic differentiation markers in C2C12 cells following siRNA-mediated knockdown of *Kazn* or *Supt3h*. (**G**) Giemsa staining revealing the myogenic differentiation of C2C12 cells after siRNA-mediated knockdown; scale bar = 100 μm. (**H**,**I**) qPCR analysis of osteogenic differentiation markers in MC3T3-E1 cells following knockdown of *Kazn* or *Supt3h*. (**J**) ALP staining assessing early osteogenic differentiation in MC3T3-E1 cells after gene knockdown. (**K**) Alizarin Red S staining evaluating matrix mineralization and late-stage osteogenic differentiation. Data are presented as mean ± SD; n = 3 per group. * *p* < 0.05; ** *p* < 0.01; *** *p* < 0.001; **** *p* < 0.0001. qPCR, quantitative PCR; ALP, alkaline phosphatase.

Notably, siRNA-mediated knockdown experiments were performed in C2C12 myoblasts and MC3T3-E1 pre-osteoblasts to functionally evaluate the roles of *KAZN* and *SUPT3H*. Efficient silencing of *Kazn* and *Supt3h* was confirmed by quantitative PCR (qPCR) in both cell types (Figure 3A–D).

In C2C12 cells, knockdown of either *Kazn* or *Supt3h* promoted myogenic differentiation, and qPCR analysis revealed increased expression of key myogenic markers, including *MyoD*, *Myogenin*, and *Desmin*, following knockdown of either gene (Figure 3E,F). Conversely, the expression of the muscle atrophy-related gene *Atrogin-1* was reduced after gene silencing. This decrease reached statistical significance in the si-*Supt3h* group, whereas a downward trend without statistical significance was observed in the si-*Kazn* group. Moreover, morphological assessment by Giemsa staining supported these findings. Compared with control cells, which exhibited limited myotube formation after differentiation induction, *Kazn*- or *Supt3h*-silenced cells exhibited enhanced myoblast fusion and increased formation of multinucleated myotubes (Figure 3G). These observations suggest that silencing either gene facilitates myogenic differentiation in vitro.

Consistent with the effects observed in myoblasts, *Kazn* or *Supt3h* knockdown enhanced osteogenic differentiation in MC3T3-E1 cells. Furthermore, qPCR analysis revealed upregulation of osteogenic markers, including *Bmp2*, *Runx2*, *Alp*, and *Ocn*, in both knockdown groups relative to the controls (Figure 3H,I). Functional staining assays further demonstrated increased alkaline phosphatase (ALP) activity and extracellular matrix mineralization after gene silencing, as indicated by stronger ALP and Alizarin Red S staining (Figure 3J,K).

Parallel control experiments confirmed the specificity of these findings. Undifferentiated cells in growth medium demonstrated minimal staining and no multinucleated myotubes or mineralized nodules, while nontransfected cells in standard differentiation medium exhibited differentiation levels comparable to the si-*NC* group, ruling out the nonspecific effects of transfection reagents. IGF-1 and BMP-2 treatments robustly enhanced myogenic and osteogenic differentiation, respectively, validating the reliability of our experimental systems (Figure S2).

Collectively, these in vitro experimental observations preliminarily suggest the tentative hypothesis that *KAZN* and *SUPT3H* function as potential negative modulators of myogenic and osteogenic differentiation under cell culture conditions.

### 2.4. Immune–Mitochondrial Programs and Immune Infiltration Patterns Associated with KAZN and SUPT3H

After confirming the functional effects of *KAZN* and *SUPT3H* in lineage differentiation, we next explored the biological programs associated with these genes in disease datasets. Gene set enrichment analysis (GSEA) was performed for *KAZN* and *SUPT3H* in the sarcopenia and osteoporosis datasets.

**Figure 4:**
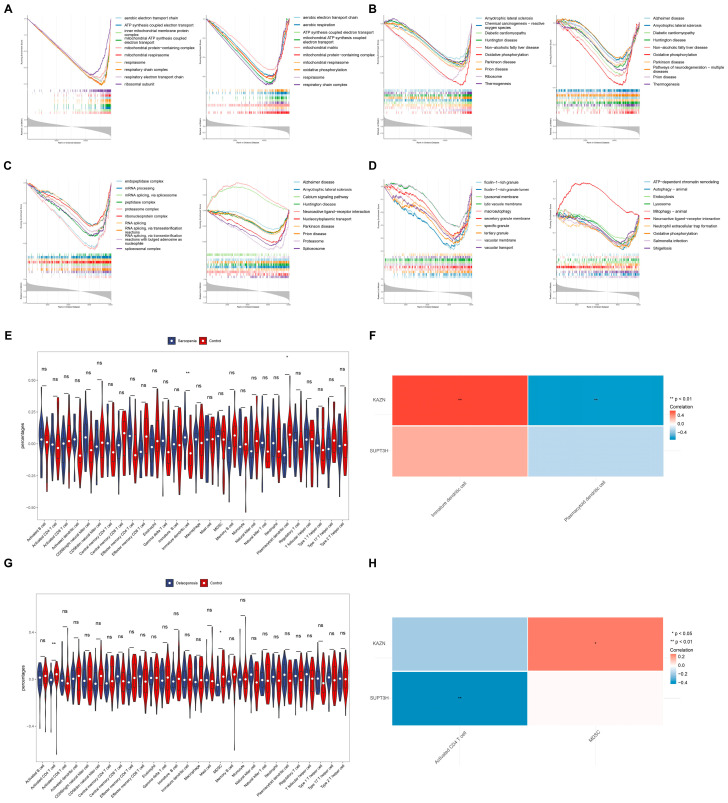
Functional divergence and immune associations of *KAZN* and *SUPT3H* across sarcopenia and osteoporosis. (**A**) GSEA-GO results for *KAZN* (left) and *SUPT3H* (right) in the sarcopenia dataset GSE111016. (**B**) GSEA-KEGG results for *KAZN* (left) and *SUPT3H* (right) in GSE111016. (**C**) GSEA-GO (left) and GSEA-KEGG (right) results for *KAZN* in the osteoporosis dataset GSE56815. (**D**) GSEA-GO (left) and GSEA-KEGG (right) results for *SUPT3H* in GSE56815. (**E**) Violin plots comparing PLAGE-inferred immune cell infiltration scores between sarcopenia and control samples. ^ns^ not significant; * *p* < 0.05; ** *p* < 0.01. (**F**) Correlation heatmap between key-gene expression and differentially abundant immune cell types in GSE111016. (**G**) Violin plots comparing immune infiltration scores between the low-BMD and high-BMD groups in GSE56815. ^ns^ not significant; * *p* < 0.05; ** *p* < 0.01. (**H**) Correlation heatmap between key-gene expression and differentially abundant immune cell types in GSE56815. GSEA, gene set enrichment analysis; GO, gene ontology; KEGG, Kyoto Encyclopedia of Genes and Genomes; BMD, bone mineral density.

In the sarcopenia dataset GSE111016, GO enrichment for genes correlated with *KAZN* and *SUPT3H* underscored mitochondrial-related processes, including mitochondrial ATP synthesis-coupled electron transport (Figure 4A). Furthermore, KEGG analysis identified enrichment in amyotrophic lateral sclerosis-related pathways (Figure 4B). These findings support the involvement of mitochondrial and degenerative stress-related programs in sarcopenic muscles.

In the osteoporosis dataset GSE56815, *KAZN*-associated genes were enriched for RNA processing-related GO terms and mapped to amyotrophic lateral sclerosis-related pathways (Figure 4C). Conversely, *SUPT3H*-associated signatures more prominently implicated immune-related pathways, including chemokine signaling and cytokine–cytokine receptor interaction (Figure 4D). Supplementary analyses further supported cross-disease consistency, with *KAZN* repeatedly linked to mitochondrial-related pathways and *SUPT3H* repeatedly linked to immune-related pathways (Tables S3–S6). Collectively, *KAZN* and *SUPT3H* participate in distinct biological cascades during musculoskeletal aging. *KAZN* is tightly linked to mitochondrial homeostasis and cytoskeleton remodeling, while *SUPT3H* predominantly mediates immune signaling and epigenetic transcriptional regulation. Given their non-overlapping functional programs, simultaneous suppression of both genes may exert additive or synergistic effects on myogenic and osteogenic differentiation, although this hypothesis remains untested in the current study.

Given the immune-related signals observed in the enrichment analyses, the immune infiltration scores for 28 immune cell types were estimated using the PLAGE algorithm. In the sarcopenia dataset, violin plots showed significant differences in dendritic cell subsets between the patients and controls (Figure 4E). Correlation analysis further indicated that *KAZN* was associated with differentially infiltrated dendritic cell subsets, including a positive correlation with immature dendritic cells and a negative correlation with plasmacytoid dendritic cells (Figure 4F). In the osteoporosis dataset, immune infiltration analysis identified differential abundance of cell types including activated CD4^+^ T cells and myeloid-derived suppressor cells between low- and high-BMD groups (Figure 4G). Correlation analysis showed that *SUPT3H* was negatively correlated with activated CD4^+^ T-cell infiltration, whereas its association with myeloid-derived suppressor cells was not significant (Figure 4H). After Benjamini–Hochberg correction, the key correlations remained significant (Tables S7 and S8).

Collectively, these results suggest that *KAZN* and *SUPT3H* are associated with distinct but potentially complementary biological programs. GSEA analyses imply that *KAZN* may correlate with mitochondrial functional programs, while *SUPT3H* may be associated with immune signaling pathways. Both genes also showed a potential association with immune microenvironment alterations across sarcopenia and osteoporosis datasets.

### 2.5. Single-Cell Analysis Reveals Cross-Tissue Cell-Type Specificity and Differentiation Dynamics of KAZN and SUPT3H

To define the cellular context of *KAZN* and *SUPT3H* across bone and muscle tissues, scRNA-seq datasets from osteoporosis bone marrow and aged skeletal muscle were analyzed.

**Figure 5:**
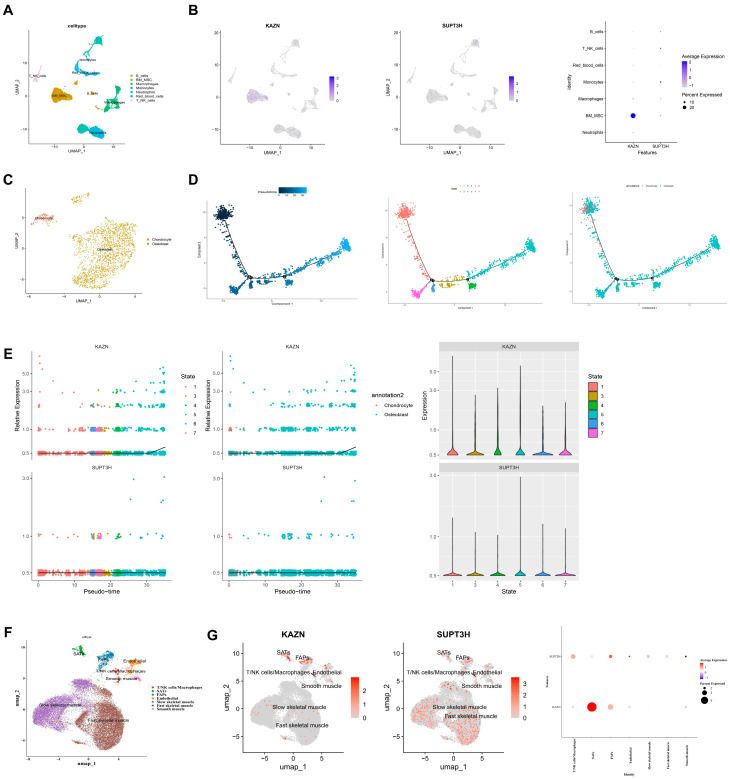
Single-cell analysis reveals cross-tissue cell-type specificity and differentiation dynamics of *KAZN* and *SUPT3H*. (**A**) UMAP visualization of annotated cell types in the osteoporosis bone marrow scRNA-seq dataset GSE147287. (**B**) UMAP feature plots and bubble plot depicting the distribution and expression of *KAZN* and *SUPT3H* across annotated cell types in GSE147287. (**C**) UMAP visualization of bone marrow mesenchymal stromal cell subclustering. (**D**) Pseudotime trajectory inference for bone marrow mesenchymal stromal cell differentiation. From left to right: pseudotime trajectory plot, cell state trajectory plot, and cell cluster trajectory plot. The numbers 1, 2, and 3 in the circle represent branch points 1, 2, and 3, respectively. (**E**) Expression dynamics of *KAZN* and *SUPT3H* along the pseudotime trajectory. The black line represents the smoothed fitted trend of gene expression across cells along pseudotime progression. (**F**) UMAP visualization of annotated cell types in the aged skeletal muscle scRNA-seq dataset GSE167186. (**G**) Cell-type-specific expression of *KAZN* and *SUPT3H* visualized using UMAP feature plots and dot plots in GSE167186. UMAP, uniform manifold approximation and projection.

In the osteoporosis bone marrow dataset GSE147287, unsupervised clustering identified multiple immune and stromal cell populations, including B cells, neutrophils, monocytes, macrophages, red blood cells, T/NK cells, and bone marrow mesenchymal stromal cells. These cell populations were annotated based on canonical markers and SingleR-assisted annotation (Figure 5A; Figure S3). Mapping of key-gene expression showed that *KAZN* was predominantly enriched in bone marrow mesenchymal stromal cells, whereas *SUPT3H* showed relatively low expression across annotated cell types (Figure 5B).

Furthermore, additional analyses supported the central role of bone marrow mesenchymal stromal cells within the osteoporosis bone marrow microenvironment. Cell–cell communication analysis also revealed extensive interaction networks, with bone marrow mesenchymal stromal cells depicting high interaction numbers and strengths with other cell populations (Figure S4). Major signaling pathways mediating these interactions included CXCL, MK, MIF, and SPP1 networks (Figure S5), which are related to inflammation, stromal regulation, and tissue remodeling. Moreover, functional enrichment across annotated cell types indicated that bone marrow mesenchymal stromal cells were involved in immune- and metabolism-related processes, including inflammatory response, steroid hormone biosynthesis, and myogenesis-related programs (Figures S6 and S7).

Given the central role of bone marrow mesenchymal stromal cells in skeletal homeostasis, subclustering and trajectory analysis were performed within this compartment. Pseudotime inference identified the differentiation trajectories toward osteogenic and chondrogenic lineages (Figure 5C,D). *KAZN* expression increased progressively along the inferred differentiation trajectory, suggesting potential involvement in osteogenic-lineage progression, whereas *SUPT3H* remained relatively stable without obvious dynamic changes (Figure 5E).

To extend these findings to skeletal muscle, the aged muscle single-cell dataset GSE167186 was analyzed. Multiple muscle-resident cell populations were identified, including satellite cells, fibroadipogenic progenitors, endothelial cells, and immune-related cells (Figure 5F). Cell-type-specific mapping demonstrated that *KAZN* expression was enriched in satellite cells and fibroadipogenic progenitors, whereas *SUPT3H* was preferentially expressed in fibroadipogenic progenitors and immune-related populations (Figure 5G).

Collectively, these single-cell analyses reveal distinct but complementary cellular distributions of *KAZN* and *SUPT3H* across bone and muscle tissues. *KAZN* appears to be associated mainly with stromal and progenitor compartments and shows dynamic expression along differentiation trajectories, whereas *SUPT3H* shows broader expression in stromal and immune-related compartments.

### 2.6. Regulatory Networks and Candidate Compound Prediction

To investigate potential upstream regulatory mechanisms and generate testable hypotheses for pharmacological modulation, multilayer regulatory networks and compound screening were performed.

**Figure 6:**
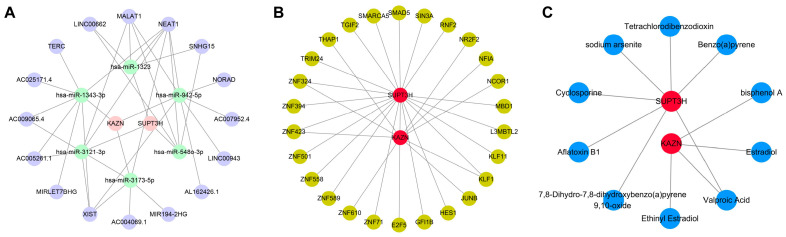
Regulatory network reconstruction and CTD-based candidate compound screening for *KAZN* and *SUPT3H*. (**A**) ceRNA network integrating lncRNAs, miRNAs, and key genes. (**B**) Transcription factor–gene regulatory network inferred from ENCODE ChIP-seq evidence. (**C**) Compound–gene interaction network derived from the CTD. CTD, Comparative Toxicogenomics Database; ceRNA, competing endogenous RNA; lncRNA, long noncoding RNA; miRNA, microRNA; ChIP-seq, chromatin immunoprecipitation sequencing.

First, a long noncoding RNA (lncRNA)–microRNAs (miRNA)–messenger RNA (mRNA) competing endogenous RNA (ceRNA) network was constructed. A total of 39 miRNAs predicted to target *KAZN* and *SUPT3H* were retrieved from the miRDB, miRWalk, and microT databases. In addition, 48 experimentally supported lncRNAs with crosslinking immunoprecipitation (CLIP) evidence were obtained from StarBase. Integration of these interactions yielded a ceRNA network comprising 24 nodes and 37 edges (Figure 6A). Topological analysis highlighted two prominent hub axes, NEAT1–hsa-miR-1343-3p–*KAZN* and NEAT1–hsa-miR-1323-5p–*SUPT3H*, suggesting a NEAT1-centered post-transcriptional regulatory architecture potentially shared by both genes.

In parallel, a transcription factor–mRNA regulatory network was constructed using ENCODE chromatin immunoprecipitation sequencing (ChIP-seq) evidence. This network contained 28 nodes and 29 edges (Figure 6B). The analysis suggested that KAZN is regulated by nine transcription factors, whereas SUPT3H is regulated by 20 transcription factors, indicating partially distinct upstream transcriptional regulatory landscapes.

Finally, to identify existing small molecules potentially related to the key genes, the Comparative Toxicogenomics Database (CTD) was queried. Ten candidate compounds were obtained, and the resulting compound–gene interaction network comprised 12 nodes and 11 edges (Figure 6C). Valproic acid was predicted to interact with *KAZN* and *SUPT3H*, nominating it as a candidate compound for future experimental evaluation.

## 3. Discussion

Herein, we integrated cross-tissue transcriptomic profiling, genetic inference, in vitro validation, immune deconvolution, single-cell analysis, regulatory network reconstruction, and compound prediction to investigate the shared candidate regulators of osteosarcopenia. Our exploratory study screened 122 overlapping DEGs from two transcriptomic datasets. Two-sample MR analysis tentatively proposed *KAZN* and *SUPT3H* as genes carrying suggestive genetic signals linked to osteoporosis risk, which cannot be interpreted as definitive causal evidence. Both genes were upregulated in the disease groups and showed weak between-group expression discrimination trends in the training datasets. Importantly, siRNA-mediated knockdown experiments demonstrated that *Kazn* and *Supt3h* suppression promoted myogenic and osteogenic differentiation, supporting their potential role as negative regulators of lineage differentiation. Subsequent pathway, immune, and single-cell analyses placed these genes within mitochondrial, immune, stromal, and progenitor-related contexts.

A key feature of this study was the use of the following distinct but biologically relevant tissues: skeletal muscle for sarcopenia and PBMCs for osteoporosis. Although this cross-tissue design inevitably introduces tissue-specific heterogeneity, it is compatible with the view of osteosarcopenia as a systemic geriatric syndrome influenced by inflammation, endocrine dysregulation, metabolic imbalance, and altered inter-organ communication [7,8,9]. PBMCs capture systemic immune and inflammatory states that may influence muscle and bone homeostasis [16]. Therefore, shared DEGs between skeletal muscle and PBMCs may reflect systemic disease-related processes rather than purely tissue-intrinsic alterations. The addition of MR further strengthened the prioritization of candidate genes by reducing the likelihood that the selected genes merely reflected downstream transcriptional consequences.

The functional validation results are specifically relevant to the biological interpretation of *KAZN* and *SUPT3H*. Silencing either *Kazn* or *Supt3h* enhanced myogenic differentiation in C2C12 cells, as indicated by increased *MyoD*, *Myogenin*, and *Desmin* expression and improved myotube formation. Likewise, knockdown of either gene promoted osteogenic differentiation and mineralization in MC3T3-E1 cells, as demonstrated by increased expression of *Bmp2*, *Runx2*, *Alp*, and *Ocn* and stronger ALP and Alizarin Red S staining. These concordant effects across muscle and osteogenic cell models suggest that *KAZN* and *SUPT3H* restrain lineage differentiation programs. This is consistent with their upregulation in disease groups, although the precise mechanisms by which they regulate differentiation require further study.

*KAZN* encodes a cytoskeleton-associated protein implicated in adhesion, migration, intracellular trafficking, and cellular architecture [17]. Studies have linked kazrin-related proteins to desmosome-associated structures, microtubule organization, and epithelial integrity [18,19]. Cytoskeletal remodeling is fundamental for myoblast fusion and osteoblast differentiation, suggesting that *KAZN* influences musculoskeletal homeostasis through cytoskeletal or mechanotransduction-related mechanisms [20]. In the present study, *KAZN* was repeatedly associated with mitochondrial-related processes in enrichment analyses and was enriched in stromal or progenitor-like compartments in single-cell datasets. These findings suggest that *KAZN* couples cellular architecture, metabolic state, and differentiation capacity in aging muscles and bones.

*SUPT3H* is a component of the SAGA histone acetyltransferase complex, which participates in chromatin organization and transcriptional regulation [21,22,23]. Epigenetic regulation is essential for lineage commitment and maintenance in muscle and bone [24]. The present results showed that *Supt3h* knockdown enhanced myogenic and osteogenic differentiation, suggesting that *SUPT3H* participates in transcriptional programs that restrain lineage progression under certain conditions. In enrichment analyses, *SUPT3H*-associated signatures were more prominently linked to immune-related pathways, including chemokine and cytokine signaling. This finding suggests that *SUPT3H* also participates in immune-regulatory transcriptional programs relevant to osteoporosis and systemic musculoskeletal aging.

Immune infiltration analysis supported gene–immune associations in both disease contexts. *KAZN* expression was associated with dendritic cell subset alterations in sarcopenia, whereas *SUPT3H* was associated with activated CD4^+^ T-cell patterns in osteoporosis. These associations do not establish direct causality between the genes and immune cell abundance but indicate that their expression may be linked to immune microenvironmental states. Given the central role of chronic low-grade inflammation in aging, these findings support inflammation as a shared contributor to bone and muscle decline [25].

An additional observation was the enrichment of neurodegeneration-related pathways, particularly amyotrophic lateral sclerosis-related signatures, among shared or gene-associated pathways. This enrichment should not be interpreted as evidence of neuron-specific pathology in osteosarcopenia. Rather, it may reflect conserved stress-response programs shared across degenerative conditions, including mitochondrial dysfunction, oxidative stress, proteostasis imbalance, inflammation, and impaired cellular repair [26,27,28,29,30,31,32]. These processes are relevant to muscle wasting, skeletal remodeling, and aging-related tissue dysfunction.

Single-cell analyses provided a cellular context for prioritized genes. *KAZN* was enriched in bone marrow mesenchymal stromal cells and showed increasing expression along inferred differentiation trajectories, while in aged skeletal muscle, it was detected in satellite cells and fibroadipogenic progenitors. These compartments are closely related to tissue repair, regeneration, and remodeling. *SUPT3H* showed a broader distribution in stromal and immune-related cell populations, consistent with its enrichment in immune-related pathways. These patterns support the hypothesis that *KAZN* and *SUPT3H* may act within complementary cellular contexts across the bone–muscle axis.

Regulatory network analyses further generated mechanistic hypotheses. The NEAT1-centered ceRNA modules involving *KAZN* and *SUPT3H* suggest possible post-transcriptional regulation, while transcription factor networks indicate partially distinct upstream transcriptional control. Compound prediction identified valproic acid as a candidate compound linked to both genes. Given the known chromatin-modifying properties of valproic acid [33] and its effects on cytoskeletal and transcriptional programs [34], this prediction is biologically plausible. However, the compound analysis was based on database-derived interactions and should be regarded as hypothesis-generating rather than evidence of therapeutic efficacy.

Several limitations should be acknowledged. First, the cross-tissue design may introduce tissue-specific noise, and matched multi-tissue samples from the same individuals would strengthen future analyses. We initially performed DEG screening using two training cohorts (GSE111016, GSE56815). To improve reproducibility, we supplemented two independent Gene Expression Omnibus (GEO) datasets: GSE167186 for sarcopenia muscle (tissue-matched) and GSE249471 for osteoporosis BMSCs (disease-relevant cell type). *KAZN* upregulation was successfully replicated in the sarcopenia muscle cohort, while *SUPT3H* showed a consistent but nonsignificant upward trend; in the osteoporosis BMSC cohort, both genes showed directionally consistent but nonsignificant upregulation. Nevertheless, all transcriptomic data in this study are retrospective public datasets without matched multi-tissue samples from the same individuals, and the osteoporosis BMSC validation cohort has a very small sample size and limited statistical power. No prospective clinical cohort was included to further verify our transcriptomic observations. Independent validation in additional large-scale, well-phenotyped cohorts is required. Second, the diagnostic ROC analysis has notable limitations. The primary ROC curves were generated using the same training datasets for DEG screening, leading to inflated AUC values caused by internal fitting bias. External validation in independent cohorts revealed unstable discriminatory effects across different tissue types and populations. The osteoporosis BMSC validation cohort showed relatively high AUC estimates, but these values should be interpreted with extreme caution due to the very small sample size and limited statistical power. Moreover, AUC values of 0.6–0.7 only represented marginal discrimination trends and failed to meet the standard for clinical biomarkers. Collectively, ROC analysis serves only as auxiliary evidence of transcriptional differences and cannot support the clinical diagnostic value of *KAZN* and *SUPT3H*. Third, our two-sample MR analysis has limitations. Only 4 and 5 eligible cis-eQTL SNPs were available for *KAZN* and *SUPT3H*, respectively, compromising MR-Egger reliability. These eQTLs were derived from peripheral whole blood and reflect the expression levels of immune-related genes in the systemic circulation, which aligns with the research context of this study—osteoporosis PBMC samples and systemic skeletal–muscular aging—limiting inference on local regulatory mechanisms. Colocalization analysis did not support shared causal variants, indicating potential linkage disequilibrium confounding [35]. Thus, MR results provide preliminary genetic evidence rather than definitive causal proof. Fourth, pseudotime analysis reflects inferred transcriptional progression rather than true chronological differentiation. Finally, although in vitro experiments supported the negative regulatory roles of *Kazn* and *Supt3h* in differentiation, further mechanistic studies and in vivo validation are needed to determine their roles in osteosarcopenia pathogenesis. In our next steps, dual siRNA cotransfection assays targeting *KAZN* and *SUPT3H* will be implemented in C2C12 and MC3T3-E1 cells to explore whether combined inhibition produces stronger prodifferentiation effects compared with single-gene knockdown, which will clarify the interactive regulatory relationship between mitochondrial and immune pathways in osteosarcopenia progression.

In summary, our in vitro data demonstrate that individual depletion of *KAZN* or *SUPT3H* relieves the inhibition of myogenic and osteogenic differentiation, generating verifiable mechanistic hypotheses that these genes may serve as potential shared modulators in osteosarcopenia pathogenesis. However, future co-knockdown experiments are warranted to elucidate the potential synergistic effects between these two distinct negative regulators. Collectively, this exploratory integrative analytical and experimental work supports a model in which mitochondrial-associated and immune-regulatory programs may converge with impaired myogenic and osteogenic differentiation to contribute to bone–muscle dysfunction. These findings provide candidate biomarkers and mechanistic hypotheses for future studies but require further validation before clinical translation.

## 4. Materials and Methods

### 4.1. Overall Workflow and Data Availability

**Figure 7:**
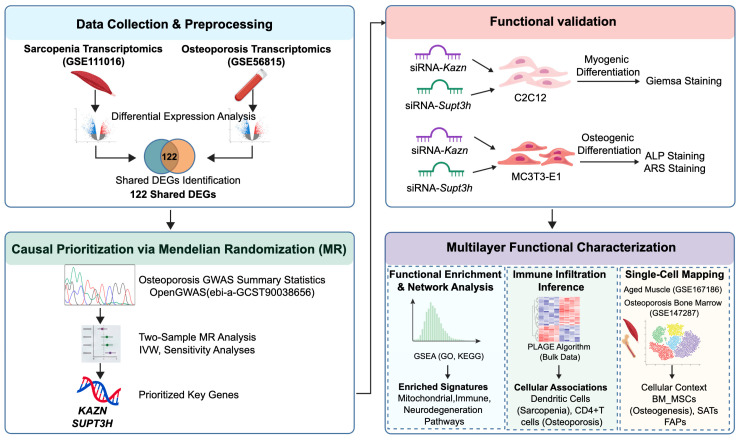
Overall workflow of this study.

This study applied an integrative framework to identify shared candidate regulators of osteosarcopenia by combining bulk transcriptomics, two-sample MR, diagnostic evaluation, in vitro validation, functional enrichment, immune infiltration inference, single-cell transcriptomics, regulatory network reconstruction, and compound prediction. Bulk and single-cell transcriptomic datasets were retrieved from the GEO database (https://www.ncbi.nlm.nih.gov/geo/, accessed on 28 January 2024). Osteoporosis genome-wide association study (GWAS) summary statistics were obtained from OpenGWAS (https://opengwas.io/, accessed on 18 February 2024). The overall workflow is summarized in Figure 7. Since all analyses were performed using publicly available, de-identified datasets, no additional ethical approval or informed consent was required. All analytical scripts for differential expression analysis, two-sample MR pipeline, GSEA, single-cell RNA-seq processing and regulatory network construction have been deposited in the public Zenodo repository (https://doi.org/10.5281/zenodo.21093053).

### 4.2. Bulk Transcriptomic Datasets

For sarcopenia, bulk RNA-seq data were obtained from GSE111016, generated on platform GPL16791 using human skeletal muscle biopsies from the vastus lateralis. The dataset included 20 sarcopenia patients and 20 controls. For osteoporosis, microarray expression data were obtained from GSE56815, generated on platform GPL96 using PBMCs. This dataset included 40 low-BMD and 40 high-BMD women. For external validation of gene expression trends, this study additionally downloaded two independent public transcriptomic datasets—the sarcopenia bulk RNA sequencing dataset GSE167186, platform GPL20301 (included 24 sarcopenia patients and 29 controls), and the osteoporosis BMSC dataset GSE249471, platform GPL24676 (included three participants with osteoporosis and three participants with osteoarthritis). To avoid analytical bias, the validation dataset was processed using standardization and differential analysis workflows that were identical to those used for the training cohort.

### 4.3. Differential Expression Analysis and Identification of Shared DEGs

Differential expression analysis was conducted separately for the sarcopenia and osteoporosis datasets. For the RNA-seq dataset GSE111016, DEGs were identified using DESeq2 (v1.38.0) [36]. For the microarray dataset GSE56815, DEGs were identified using limma (v3.54.0) [37]. To capture a broad set of candidate genes for downstream integration, genes with *p* < 0.05 were considered differentially expressed in each dataset [38,39].

Shared candidate genes were defined as the intersection of DEGs from the two datasets and were used for subsequent comorbidity analyses.

### 4.4. Functional Enrichment of Shared DEGs

GO and KEGG enrichment analyses of shared DEGs were performed using clusterProfiler (v4.7.1.3) [40]. Enrichment terms with *p* < 0.05 and gene count > 1 were considered significant.

### 4.5. Two-Sample MR

#### 4.5.1. GWAS Outcome Data

Summary statistics for osteoporosis were obtained from OpenGWAS using the GWAS ID ebi-a-GCST90038656. The dataset included 484,598 cases and 476,847 controls with 9,587,836 SNPs.

#### 4.5.2. Instrument Selection

Genetic instruments were constructed using the cis-eQTL obtained from OpenGWAS. All cis-eQTL instrumental SNPs were derived from whole blood tissue. SNPs associated with gene expression at genome-wide significance were retained using *p* < 5 × 10^−8^. Linkage disequilibrium clumping was performed using PLINK (v1.9) with *r*^2^ < 0.001 and a 10,000-kb window. SNPs with weak instrument strength, defined as *F* < 10, were excluded.

#### 4.5.3. MR Implementation and Sensitivity Analyses

Two-sample MR analyses were performed using the TwoSampleMR R package (v0.5.6) [41]. Exposure and outcome summary statistics were harmonized to ensure consistent alignment of effect alleles. The primary causal effect was estimated using the IVW fixed-effects model. Sensitivity analyses were performed using MR-Egger, weighted median, and simple median methods.

Robustness was further evaluated using heterogeneity tests, horizontal pleiotropy tests, and leave-one-out analyses. Directionality was assessed using the Steiger directionality test.

### 4.6. Definition of Key Genes and Diagnostic Performance

Genes supported by MR evidence were defined as key genes. ROC analysis was performed using the pROC R package (v1.18.0) [42] to evaluate the diagnostic performance of key genes in both bulk datasets.

### 4.7. Cell Culture

Mouse C2C12 myoblasts (CL-0044) and MC3T3-E1 subclone 14 pre-osteoblasts (CL-0378) were obtained from Procell Biotechnology Co., Ltd. (Wuhan, China). C2C12 cells were maintained in high-glucose DMEM (Thermo Fisher Scientific, Waltham, MA, USA, C11995500BT) supplemented with 10% fetal bovine serum (FBS; Bio-Channel Biotechnology, Nanjing, China, BC-SE-FBS01C) and 1% penicillin–streptomycin. MC3T3-E1 cells were cultured in α-MEM (Procell Life Science & Technology, Wuhan, China, PM150421) containing 10% FBS and 1% penicillin–streptomycin. Cells were maintained at 37 °C in 5% CO_2_, routinely tested for mycoplasma contamination, and used between passages 3 and 12.

### 4.8. siRNA Transfection

siRNAs targeting *Kazn* and *Supt3h* were procured from IGE Biotechnology Ltd. (Guangzhou, China). Cells were plated in 12-well plates and transfected using Lipofectamine™ RNAiMAX (Invitrogen, Carlsbad, CA, USA, 13778150) and Opti-MEM Reduced Serum Medium (Thermo Fisher Scientific, Waltham, MA, USA, 31985070) according to the manufacturer’s instructions. Furthermore, the siRNA dose was 0.2 nmol per 1 × 10^5^ cells. The medium was replaced after 5 h, and knockdown efficiency was assessed after 48 h. The siRNA sequences were as follows: si-*NC*: 5′-UUCUCCGAACGUGUCACGUTT-3′; si-*Kazn*-1879: 5′-CCAAGAAGUUCCACCAAGUUA-3′; si-*Kazn*-1256: 5′-CCCUAUUGUACAGUCACUAGA-3′; si-*Kazn*-2376: 5′-CCCGAUUUCCAUGAUGACUAU-3′; si-*Supt3h*-294: 5′-CACUUCAAGUAGUGGAAGAAA-3′; si-*Supt3h*-281: 5′-CGUACUAUGGAUUCAGCUCAA-3′; si-*Supt3h*-964: 5′-GCUCUUCUAGUGAGGCAAGAU-3′.

### 4.9. RNA Extraction and qPCR

Total RNA was extracted using an RNA extraction kit according to the manufacturer’s instructions (ESscience, Shanghai, China, RN001). RNA concentration and purity were assessed using a NanoDrop 2000 spectrophotometer (Thermo Fisher Scientific, Waltham, MA, USA). RNA was reverse-transcribed into cDNA using PrimeScript RT Master Mix (TaKaRa, Otsu, Japan, RR036A) under the following conditions: 37 °C for 15 min, 85 °C for 5 s, and 4 °C for 5 min. qPCR was performed using a Roche LightCycler 96 system (Roche, Indianapolis, IN, USA). Moreover, GAPDH was used as the internal control, and relative gene expression was calculated using the 2^−ΔΔCt^ method. Primer information is provided in Table S9.

### 4.10. Myogenic Differentiation and Giemsa Staining

For myogenic differentiation, C2C12 cells were switched to a differentiation medium consisting of DMEM supplemented with 2% horse serum (Procell Life Science & Technology, Wuhan, China, 164215) when they reached approximately 80% confluence. Differentiation was induced for 6 days, with medium replacement every 48 h. The cells were fixed with 4% paraformaldehyde for 15 min and stained with Giemsa solution (Jiancheng, Nanjing, China, D011-1) for 20 min. After washing, the images were captured under a light microscope. Myotube formation was qualitatively assessed based on morphology and staining intensity. For the positive control group, 10 ng/mL recombinant mouse IGF-1 (MedChemExpress, Monmouth Junction, NJ, USA, HY-P70698) was added to the differentiation medium with medium replacement every 48 h.

### 4.11. Osteogenic Differentiation, ALP Staining, and Alizarin Red S Staining

MC3T3-E1 cells were induced for osteogenic differentiation using α-MEM supplemented with 10% FBS, 1% penicillin–streptomycin, 10 mM β-glycerophosphate (Aladdin, Shanghai, China, G755749), 50 μg/mL ascorbic acid (Beyotime, Shanghai, China, Y023980), and 10 nM dexamethasone (Beyotime, Shanghai, China, Y026170). After 7 days, the cells were stained using an ALP staining kit (Beyotime, Shanghai, China, C3206) according to the manufacturer’s protocol. The staining results were visualized and captured using an inverted microscope. After 14 days of osteogenic induction, the cells were stained with Alizarin Red S working solution (Solarbio, Beijing, China, G8550) according to the manufacturer’s protocol, and mineralized nodules were observed and recorded under a microscope. For the positive control group, 50 ng/mL recombinant human BMP-2 (MedChemExpress, Monmouth Junction, NJ, USA, HY-P7006) was added to the osteogenic induction medium, with medium replacement every 3 days.

### 4.12. GSEA

To determine the biological pathways associated with key genes, GSEA was performed using clusterProfiler (v4.7.1.3). Spearman correlation coefficients between each key gene and all other genes were calculated using the psych R package (v2.2.9). Ranked gene lists were constructed based on correlation coefficients and subjected to GSEA. Terms with *p* < 0.05 were considered significant.

### 4.13. Immune Infiltration Inference

Immune cell infiltration scores for 28 immune cell types were estimated using the PLAGE algorithm [43]. Between-group differences in immune cell abundance were assessed using the Wilcoxon rank-sum test. Immune cell types showing differential abundance were correlated with key-gene expression using Spearman correlation analysis. Correlations were considered meaningful when |*r*| > 0.3 and *p* < 0.05. To control for multiple testing, *p* values were adjusted using the Benjamini–Hochberg method.

### 4.14. scRNA-Seq Analysis

The scRNA-seq dataset GSE147287, generated on platform GPL24676 and profiling bone marrow mononuclear cells from an osteoporosis patient, was included in the analysis. Quality control was performed using Seurat (v5.0.1) [44]. Cells were retained if they expressed 150–8000 genes and had less than 20% mitochondrial gene content. Highly variable genes were identified using FindVariableFeatures with the vst method and 2000 features, followed by principal component analysis. Dimensionality reduction and unsupervised clustering were performed using Seurat’s graph-based workflow. Cell type annotation was conducted using SingleR (v1.14.1) and canonical marker genes (Figure S3). Cell–cell communication analysis was performed using CellChat (v1.6.1) [45], and functional profiling of cell types was conducted using ReactomeGSA (v1.16.0). The expression of key genes was visualized using FeaturePlot (v4.4.0). Developmental trajectories were inferred using Monocle2 (v2.30.0).

The scRNA-seq dataset GSE167186, also generated on platform GPL24676, was downloaded as an aged skeletal muscle single-cell resource, including 11 aged skeletal muscle samples. Quality control was conducted using Seurat (v5.0.1) to remove low-quality cells. Cells were retained according to the following criteria: 200 < nFeature_RNA < 2000, 200 < nCount_RNA < 5000, and mitochondrial reads <25%. Genes detected in fewer than three cells were excluded. Downstream processing included variable feature selection, principal component analysis, graph-based clustering, and visualization. Clustering was performed at a resolution of 0.6, and cluster-specific marker genes were identified using FindAllMarkers (min.pct = 0.25, logfc.threshold = 0.5). Cell types were annotated using the established marker genes reported previously [46]. Dot plots and uniform manifold approximation and projection (UMAP) visualizations were generated to assess key-gene expression patterns across annotated cell populations.

### 4.15. Regulatory Network Construction

To explore post-transcriptional regulatory mechanisms involving the key genes, a ceRNA network encompassing lncRNAs, miRNAs, and mRNAs was constructed. miRNAs targeting the key genes were retrieved from the miRDB (https://mirdb.org/, accessed on 29 February 2024), miRWalk (http://mirwalk.umm.uni-heidelberg.de/, accessed on 29 February 2024), and microT (https://dianalab.e-ce.uth.gr/microt_webserver/, accessed on 29 February 2024) databases. LncRNAs interacting with these miRNAs were predicted using StarBase (http://starbase.sysu.edu.cn, accessed on 29 February 2024) with a CLIP experimental support threshold of clipExpNum ≥5. Upstream transcription factors of the key genes were predicted based on ENCODE ChIP-seq evidence integrated into the NetworkAnalyst platform (https://www.networkanalyst.ca/NetworkAnalyst/, accessed on 29 February 2024).

### 4.16. Candidate Compound Screening

Potential small-molecule compounds related to the key genes were queried from the CTD (https://ctdbase.org/, accessed on 29 February 2024). The resulting molecular interaction networks were integrated and visualized using Cytoscape (v3.7.1) [47].

## 5. Conclusions

This study integrated transcriptomic, genetic, experimental, immune, single-cell, and regulatory analyses to investigate the shared molecular features of sarcopenia and osteoporosis. *KAZN* and *SUPT3H* were identified as shared candidate genes associated with osteoporosis risk and upregulated in the disease groups. Functional experiments revealed that knockdown of *Kazn* or *Supt3h* enhanced myogenic and osteogenic differentiation in vitro, supporting their role as negative regulators of lineage differentiation. Moreover, mechanistic analyses suggest that *KAZN* is more closely related to mitochondrial and progenitor-associated programs, whereas *SUPT3H* is linked to immune and transcriptional regulatory processes. These findings provide candidate molecular targets and mechanistic hypotheses for osteosarcopenia.

## Data Availability

The data presented in this study are available in Zenodo at https://doi.org/10.5281/zenodo.21093053. These data were derived from the following resources available in the public domain: Bulk transcriptomic datasets were obtained from the GEO under accession numbers GSE111016 and GSE56815. scRNA-seq datasets were retrieved from GEO under accession numbers GSE147287 and GSE167186. Osteoporosis GWAS summary statistics were accessed via OpenGWAS (GWAS ID: ebi-a-GCST90038656).

## Supplementary Materials

The following supporting information can be downloaded at https://www.mdpi.com/article/10.3390/ijms27146340/s1.

## Abbreviations

The following abbreviations are used in this manuscript:

MR	Mendelian randomization
scRNA-seq	Single-cell RNA sequencing
PBMC	Peripheral blood mononuclear cell
DEG	Differentially expressed gene
KEGG	Kyoto Encyclopedia of Genes and Genomes
GO	Gene ontology
eQTL	Expression quantitative trait locus
IVW	Inverse variance weighted
SNP	Single-nucleotide polymorphism
BMD	Bone mineral density
ROC	Receiver operating characteristic
AUC	Area under the curve
BMSC	Bone marrow-derived mesenchymal stem cell
qPCR	Quantitative PCR
ALP	Alkaline phosphatase
GSEA	Gene set enrichment analysis
LncRNA	Long non-coding RNA
miRNA	MicroRNA
mRNA	Messenger RNA
CLIP	Cross-linking immunoprecipitation
ChIP-seq	Chromatin immunoprecipitation sequencing
CTD	Comparative Toxicogenomics Database
GEO	Gene Expression Omnibus
GWAS	Genome-wide association study
FBS	Fetal bovine serum
UMAP	Uniform manifold approximation and projection
GSVA	Gene Set Variation Analysis
