# Supplementary material for: Integrative Analysis Coupled with In Vitro Validation Reveals KAZN and SUPT3H as Shared Negative Regulators in Osteosarcopenia
Source: Int J Mol Sci. 2026 Jul 16;27(14):6340. doi: 10.3390/ijms27146340 (PMC13410197; doi:10.3390/ijms27146340)
Supplement: Supplementary file 1 [file ijms-27-06340-s001.zip › Supplementary figures and tables legend.pdf]

Supplementary Figures Legend

Figure S1. External validation of *KAZN* and *SUPT3H* expression and discriminatory performance in independent cohorts. (A) Box plots showing expression levels of *KAZN* and *SUPT3H* in sarcopenia versus control skeletal muscle samples from the GSE167186 cohort. ns, not statistically significant. (B) ROC curves evaluating the discriminatory capacity of *KAZN* and *SUPT3H* for sarcopenia in the GSE167186 cohort. (C) Box plots showing expression levels of *KAZN* and *SUPT3H* in osteoporosis versus control BMSC samples from the GSE249471 cohort. ns, not statistically significant. (D) ROC curves evaluating the discriminatory capacity of *KAZN* and *SUPT3H* for osteoporosis in the GSE249471 cohort.

Figure S2. Control groups for myogenic and osteogenic differentiation assays. (A) Giemsa staining of C2C12 cells under three control conditions: undifferentiated cells cultured in growth medium (left), non-transfected cells cultured in standard differentiation medium (middle), and cells treated with 10 ng/mL IGF-1 in differentiation medium as the positive control (right). (B) ALP staining of MC3T3-E1 cells under three control conditions: undifferentiated cells in growth medium (left), non-transfected cells in standard osteogenic induction medium (middle), and cells treated with 50 ng/mL BMP-2 as the positive control (right). (C) Alizarin Red S staining of MC3T3-E1 cells under three control conditions: undifferentiated cells in growth medium (left), non-transfected cells in standard osteogenic induction medium (middle), and cells treated with 50 ng/mL BMP-2 as the positive control (right). Scale bars, 100 μm.

Figure S3. SingleR-based cell-type annotation for osteoporosis bone marrow scRNA-seq dataset GSE147287.

Figure S4. CellChat-inferred intercellular communication networks in osteoporosis bone marrow.

Figure S5. CellChat-derived signaling pathway networks underlying intercellular communication in osteoporosis bone marrow.

Figure S6. Hallmark pathway activity across annotated bone marrow cell types estimated by Gene Set Variation Analysis (GSVA).

Figure S7. KEGG pathway activity across annotated bone marrow cell types estimated by GSVA.

Supplementary Tables Legend

Table S1. Sensitivity analyses for key genes, including heterogeneity and horizontal pleiotropy tests.

Table S2. Directionality analyses for key genes to evaluate potential reverse causation.

Table S3. Single-gene GSEA of *KAZN* for GO terms in the sarcopenia dataset GSE111016.

Table S4. Single-gene GSEA of *KAZN* for GO terms in the osteoporosis dataset GSE56815.

Table S5. Single-gene GSEA of *SUPT3H* for KEGG pathways in the sarcopenia dataset GSE111016.

Table S6. Single-gene GSEA of SUPT3H for KEGG pathways in the osteoporosis dataset GSE56815.

Table S7. Correlations between key genes and differentially infiltrated immune cell types in GSE111016.

Table S8. Correlations between key genes and differentially infiltrated immune cell types in GSE56815.

Table S9. Information on qPCR primers.
