# Supplementary figures and images for: Integrative Analysis Coupled with In Vitro Validation Reveals KAZN and SUPT3H as Shared Negative Regulators in Osteosarcopenia
Source: Int J Mol Sci. 2026 Jul 16;27(14):6340. doi: 10.3390/ijms27146340 (PMC13410197; doi:10.3390/ijms27146340)

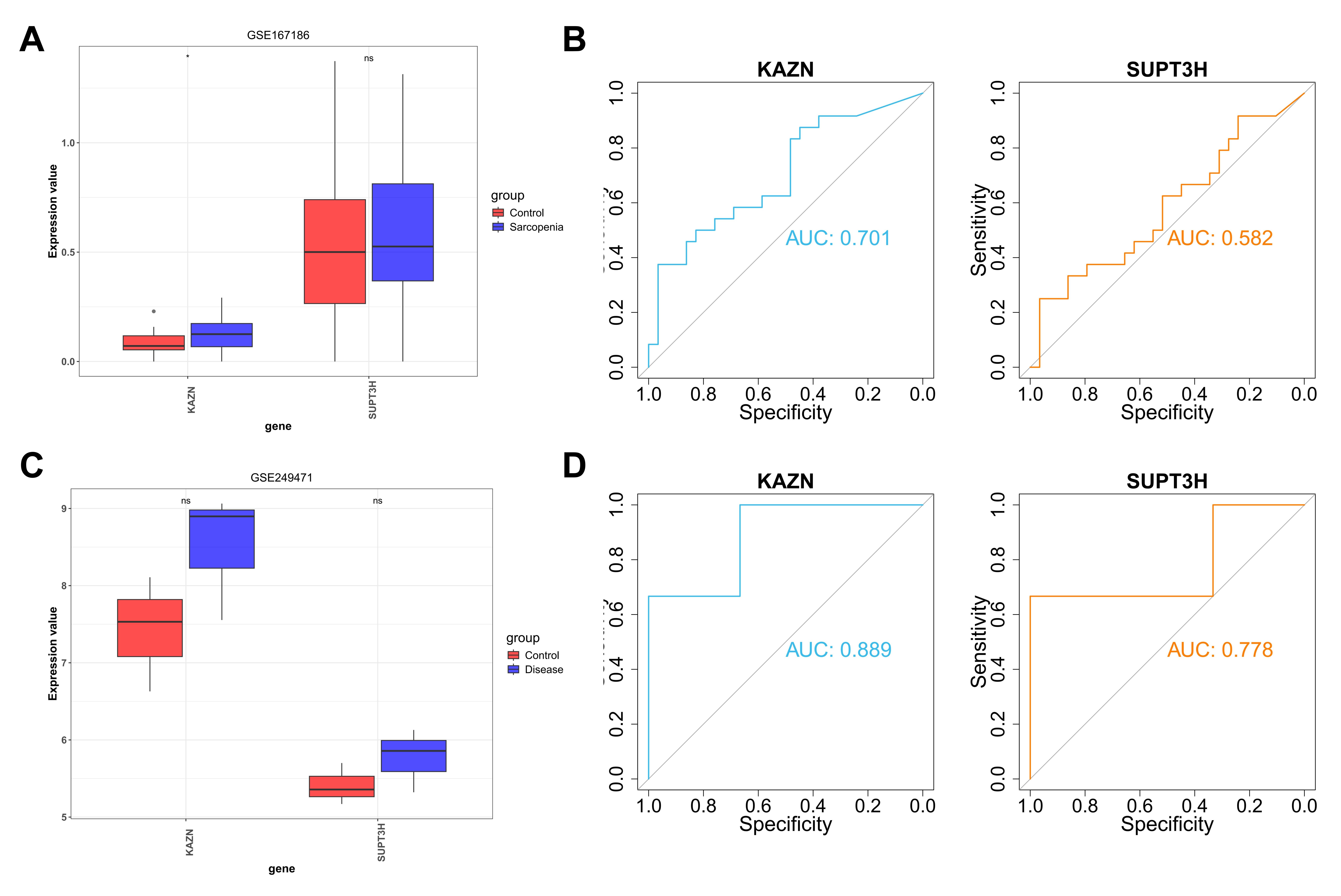

Supplement: Supplementary file 1 [file ijms-27-06340-s001.zip › Supplementary figures/Supplementary Figure S1.jpg]

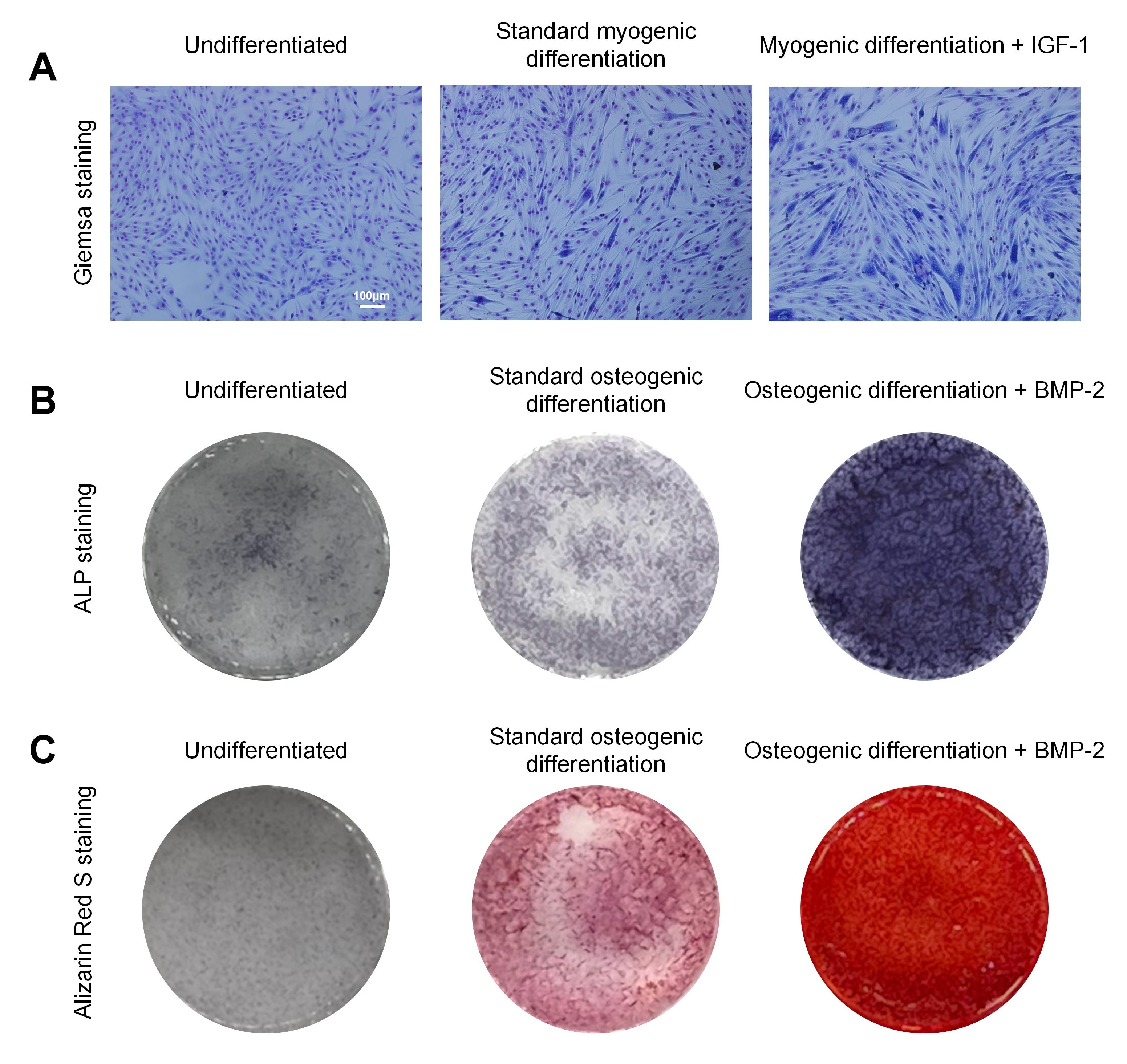

Supplement: Supplementary file 1 [file ijms-27-06340-s001.zip › Supplementary figures/Supplementary Figure S2.jpg]

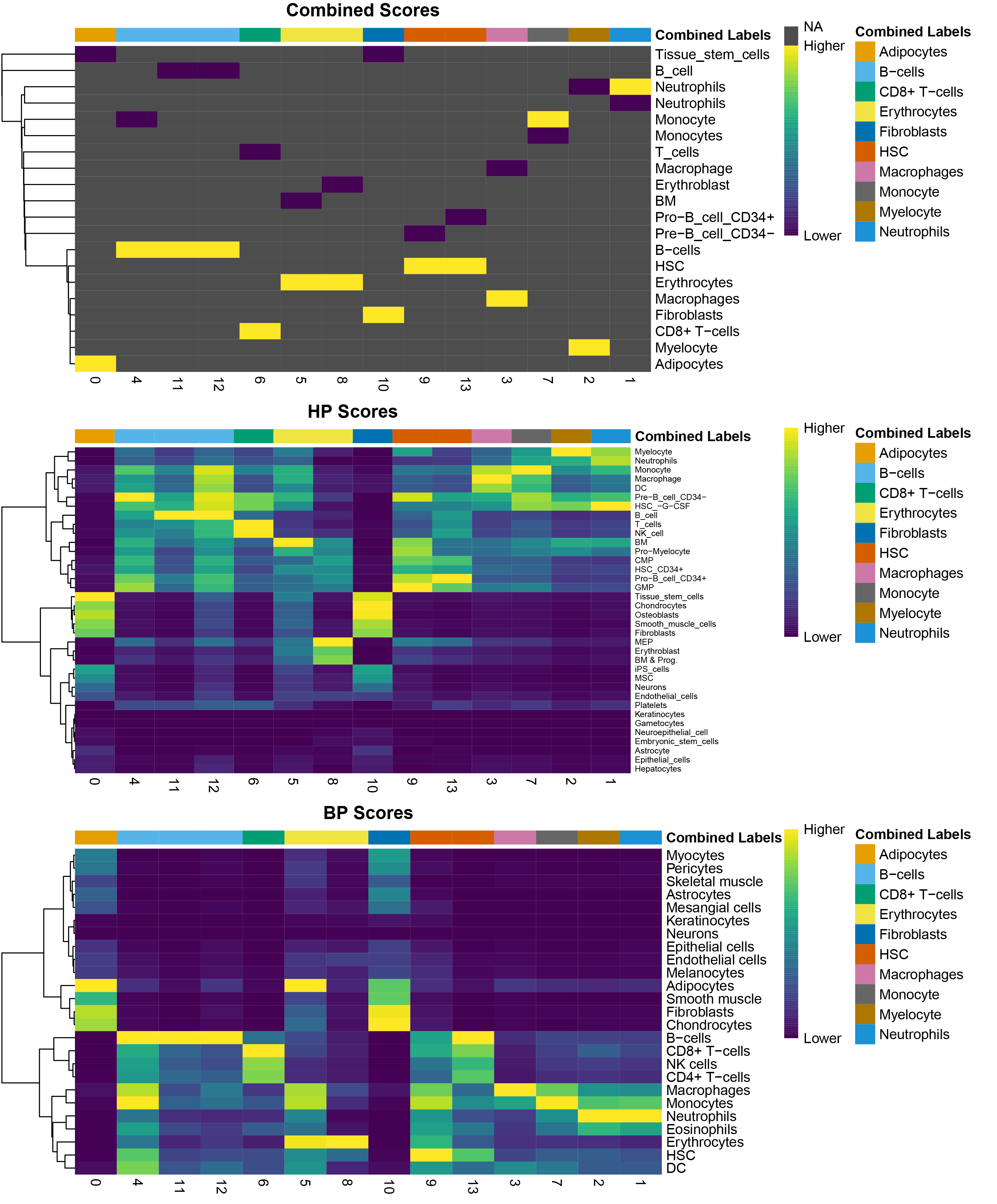

Supplement: Supplementary file 1 [file ijms-27-06340-s001.zip › Supplementary figures/Supplementary Figure S3.jpg]

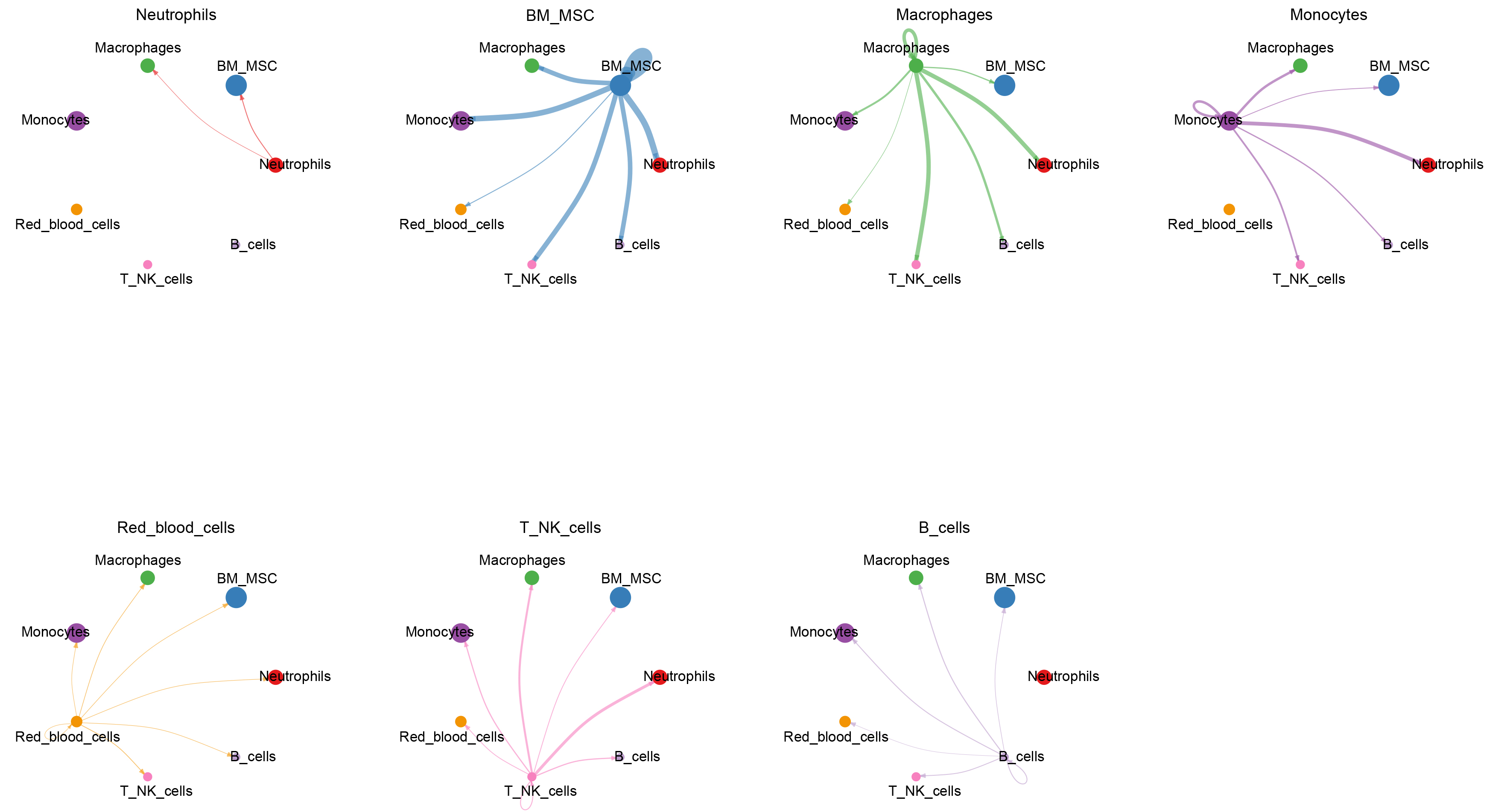

Supplement: Supplementary file 1 [file ijms-27-06340-s001.zip › Supplementary figures/Supplementary Figure S4.jpg]

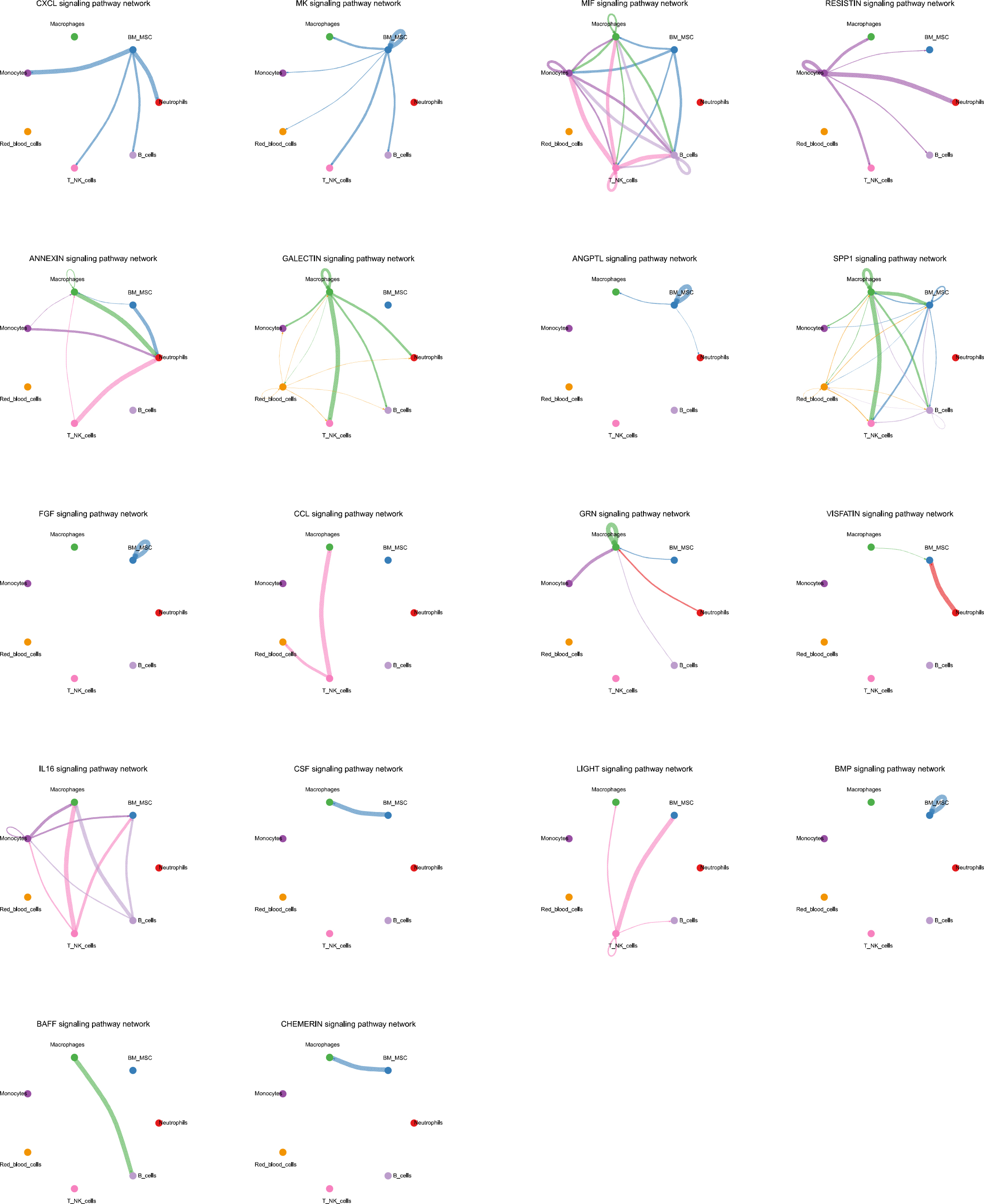

Supplement: Supplementary file 1 [file ijms-27-06340-s001.zip › Supplementary figures/Supplementary Figure S5.jpg]

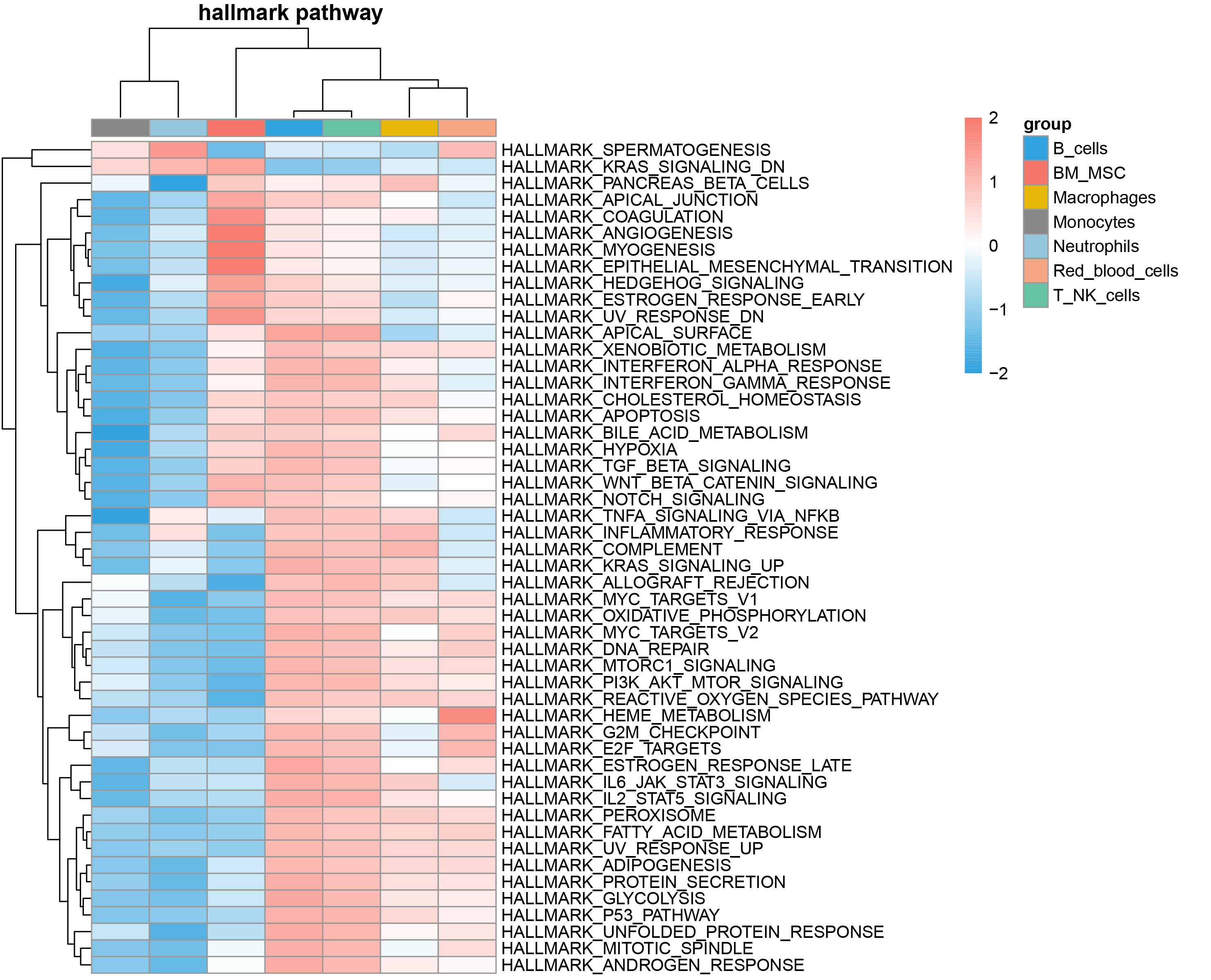

Supplement: Supplementary file 1 [file ijms-27-06340-s001.zip › Supplementary figures/Supplementary Figure S6.jpg]

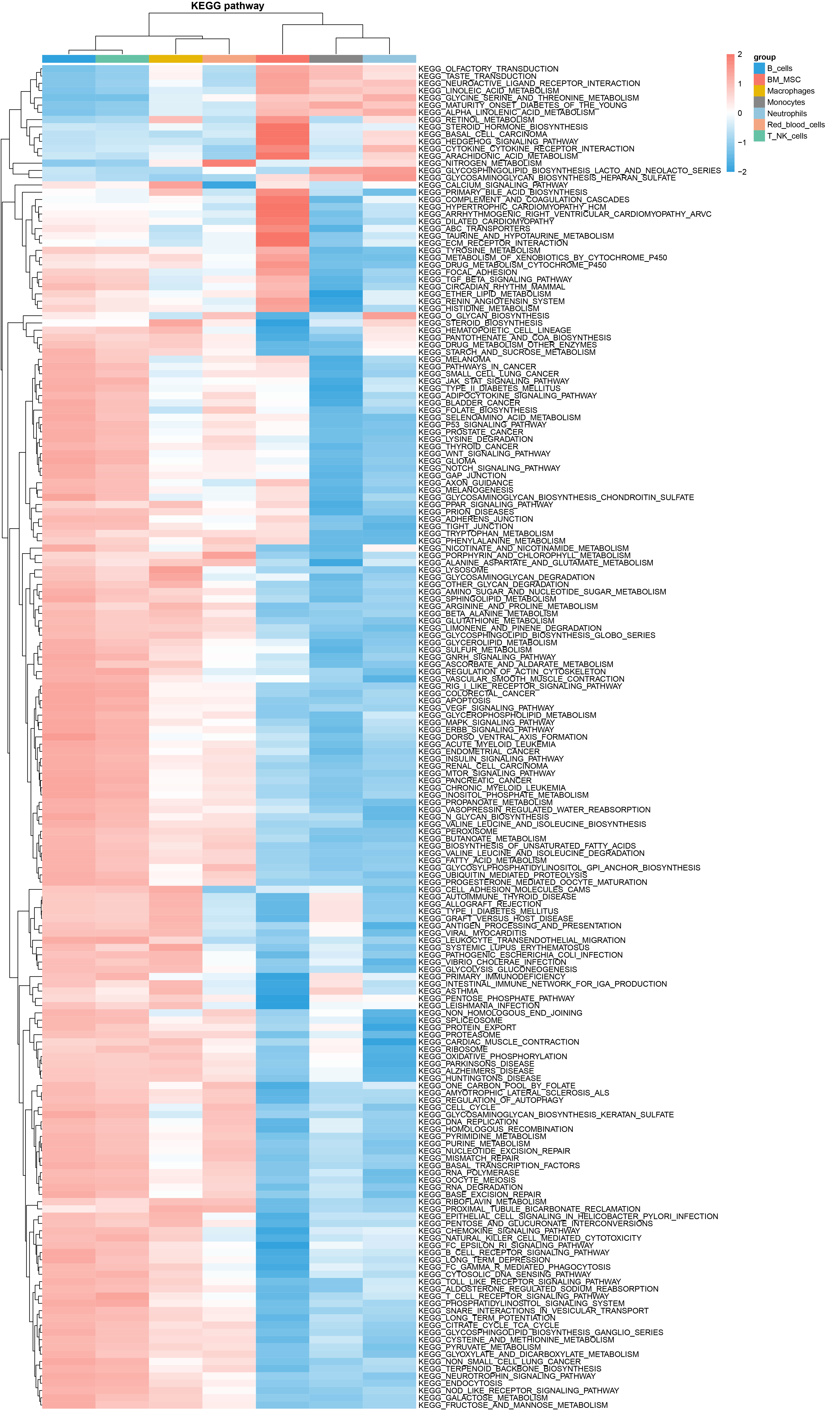

Supplement: Supplementary file 1 [file ijms-27-06340-s001.zip › Supplementary figures/Supplementary Figure S7.jpg]
